# New drug discovery for Alzheimer's disease: Challenges and hopes

**DOI:** 10.4103/0019-5545.49444

**Published:** 2009

**Authors:** T. S. Sathyanarayana Rao, K. Jagannatha Rao

**Affiliations:** Department of Psychiatry, JSS University, JSS Medical College Hospital, Ramanuja Road, Mysore - 570 004, India; 1 Department of Biochemistry and Nutrition, Central Food and Technological Research Institute, Mysore - 570 020, India

Alzheimer's disease (AD) is a common neurodegenerative disease that affects cognitive function in the elderly. It is a common dementia related to aging and a fatal neurodegenerative disease, which is on rise in both the developed and the developing countries. The disease has a complex pathology and etiology. There are no biomarkers for early detection of the disease. There are no successful drugs either to cure or to manage the disease. It has affected 4.5 million people in the United States alone in 2000 and is expected to reach about 15 million by the year 2050 in spite of all the medical advancement. Patients develop progressive cognitive decline with psychiatric and behavioral problems that impair daily living activities and also passively affect quality of life of the patient and the family. The burden of AD has an extraordinary social and economic impact on the families and on the world itself. This makes the development of potential therapies a high priority area in biomedical research. The main challenge is to choose the right biochemical target for drug discovery because of complex neuropathology of the AD brain. The large extracellular beta-amyloid (Aβ) plaques and tau-containing intraneuronal neurofibrillary tangles characterize AD from a histopathologic perspective.[1] However, the severity of dementia in AD is more closely related to the degree of the associated neuronal and synaptic loss. It is not known how neurons die and synapses are lost in AD.[2] Most of the evidence indicates that amyloid precursor protein (APP) processing has a central role in the AD process. The Aβ in the form of plaques is a metabolite of the APP that forms when an alternative (beta-secretase and then gamma-secretase enzymatic pathway) is utilized for processing. A total of six mutations have been described in the APP gene, which leads to AD by influencing APP metabolism.[3] One of the leading theories is that Aβ in plaques leads to AD because Aβ is directly toxic to the adjacent neurons. Other theories advance the notion that neuronal death is triggered by intracellular events that occur during APP processing or by extraneuronal preplaque Aβ oligomers. Now, AD has been understood as a more general problem with protein processing, from accumulation of intraneuronal Aβ or extracellular, preplaque Aβ leading to neuronal cell death. However, recently functional imaging studies implied that functional decline in humans can occur separately from both neuronal loss and neurofibrillary tangles. Aβ, a 39–43 aminoacid peptide derived from APP is the major component of senile plaques observed in AD. Evidences implicate a central role for Aβ in the pathophysiology of AD. Av40 is the dominant species in human cerebrospinal fluid, accounting for approximately 90% of total A under normal conditions. There is a disease-specific increase in Aβ40 brain tissue levels compared with the Aβ42 change observed both in the aged and in the AD brain. It is now speculated that Aβ pathological significance is also known to be associated with α-synuclein aggregation, a protein implicated in Parkinson's disease. Aβ is a self-aggregating protein and the conformational transition from unfolded state to a beta sheet-rich conformation leads to deposition of protein aggregates. Although Aβ deposits are primarily extracellular, studies have demonstrated an intraneuronal accumulation of Aβ in AD-vulnerable regions. It is said that the “amyloid is deposited first in the neuron and later in the extracellular space.” Thus, neurodegeneration (ND) includes complex pathology and etiology. No animal model completely reflects human ND seen in the brain. The major challenges are how to choose the best neuronutraceuticals for clinical trials, what kind of preclinical study design is required and whether we can formulate a synthetic or modification of the natural compound best suited for the brain. These are puzzling questions to scientists and to their brains. No tall claims can be made based on the one property of neutraceuticals and we have already learnt lessons in recent times regarding this phenomena. There are numerous studies on natural products as ND intervention molecules, but there is only limited success in them effectively reaching patients.

None of the above proved as positive success yet and have failed in many clinical trial phases. It is nearly impossible to know which of the above nutrients, herbs or combinations thereof are helpful as a treatment, prevention or alternative AD medication. It may be a trial and error process until the appropriate scientific information is obtained. There is lot of debate on curcumin and its effectiveness is in doubt because recent studies indicated that the dementia rate in India and in other parts of the world are the same. The main issue is to understand the multifunctional effect of neutraceuticals instead of targeting reduction in amyloid load, etc. The multifunctional analysis includes a molecule having the ability to enter the brain, act as an antineuroinflammatory, antioxidant, antiamyloid-tau oligomer prevention, prevention of redox iron formation, mitochondrial protectant and many more to mention. The proposed issue will detail how to optimize and target the screening of these natural products in AD intervention before their bedside use.[4] There is a plethora of publications coming up on natural products as intervention molecules in neurodegenerative disorders. However, many do not know the target of these molecules in the brain, including brain bioavailability. There is a need to formulate a novel screening program and target systems and evaluate efficacy through chemical and computation models. There is an urgent need to understand these phenomena.

Further, the clinical management of brain disorders is a challenge to physicians. At first, there is apathy in having no drug to manage neurodegenerative patients whereas, on the other hand, caregivers put pressure on alternative therapies.

The new inventions regarding the RNA interference in the management of gene expression as a possible tool in preventing or arresting ND has come to the lime light in recent times. Andrew Fire and Craig Mello won the Nobel Prize in Physiology in 2006 for discovering RNA interference (RNAi). RNA technology is of a very recent origin and won noble recognition in a very short time period, indicating its high potential in drug discovery for a number of disorders. In a PubMed search, the term “RNAi or RNA interference” showed 3500 articles in the year 2006 alone, while the number was 30 in 1999. This indicates the potential application of RNAi and its clinical application in the future.[5–7]

The major concept of RNA-based therapies is the RNA-based mechanism that silences genes in a sequence-specific manner. It is demonstrated in nematodes: very small amounts of double-stranded RNA (dsRNA) complementary to a particular gene will reduce the expression of that gene by silencing its messenger RNA (mRNA). The dsRNA-mediated silencing technology proved to be potent than that of single-stranded antisense agents. It is shown that a few dsRNA molecules per cell are sufficient to silence the targeted gene.[8]

There are mapping studies regarding the presence of specific RNAi involved in cellular processes, which are critical for brain development, neuronal differentiation and dendritic spine organization. Also, recent studies indicate that changes in RNAi are likely to play a pivotal role in neurological disorders. To cite an example, dysregulation in the RNAi pathway may play a role in various neurodevelopmental disorders. The fragile X mental retardation protein, which is deficient in fragile X syndrome, is the popular pediatric mental retardation. This is attributed to RNAi machinery. Scientists are looking for an RNA-based therapy for this.

The amyloid hypothesis is a fundamental pathological event in AD. The sequential cleavage of the transmembrane APP by BACE and γ-secretase generates toxic C-terminal fragments (CTF) and Aβ, which in turn deposits it in the extracellular space as a seed for plaque formation. Based on this assumption, attempts are made to decrease the amount of APP, BACE or γ-secretase, thereby reducing the toxic CTF and Aβ production and exert the therapeutic potential. Many drugs are targeted in this direction but no effective drug is validated to control APP pathways due to the highly complex assembly of APP.

Now, scientists are attempting to develop therapeutic RNA interference for AD. Till now, APP, BACE1 and tau have been the targets to development of RNAi-based therapies for AD. The RNAi methods are used to suppress the expression of BACE, or APP, which reduced Aβ production in cultured neurons. In other studies, it has been shown that allele-specific silencing of APP and tau is possible even in familial disorders (harboring disease-linked mutations). The first *in vivo* trial of RNAi for AD was conducted through lentiviral delivery of RNAi targeting toward endogenous BACE1 in a transgenic mouse model of AD. This mouse model overexpresses human APP, having two different disease-linked mutations. This mouse model typically develops AD-like pathology and shows congnitive deficits. When RNAi is injected for 10 months, it suppresses the expression of BACE1 both in control transgenic animals. However, in mice overexpressing mutant APP, the reduction in BACE1 levels decreased the Aβ/CTF production and thus no plaque formation is observed in the hippocampus.

These outstanding studies indicate that RNAi is promising in experimental neurotherapeutics as of now and there is an opening in the treatment of otherwise incurable diseases. But, the missing link is still the lack of clarity in the mechanisms involved. However, early lead animal studies allow us to be hopeful but cautious about the application of RNAi therapy for neurodegenerative diseases. However, one needs to conduct additional studies focusing on the efficient RNAi delivery methods to the brain and to assess any toxic effects.[9]

## THE RNA INTERFERENCE THERAPEUTIC PIPELINE: A RAY OF HOPE

There are already human phase I and II therapeutic RNAi trials on the way. These trials are targeted toward age-related macular degeneration and respiratory syncytial virus and hepatitis C infection, etc. But, there are no early trials targeted to brain disease. However scientists are opening avenues in this direction using RNAi-mediating compounds. These trials may provide an insight on potential safety issues arising from exogenous manipulation of intrinsic RNAi pathways in humans.

The brain is unique, both anatomically and neurochemically. Hence, targeting the central nervous system diseases poses additional hurdles for the ongoing RNAi trials. The biggest challenge faced by the field of RNAi research is to achieve a safe and efficient delivery of therapeutic RNAi agents to specific neurons protected by the blood–brain barrier. The other challenge is sustained delivery of an RNAi reagent by repeated administration for neurodegenerative disorder to achieve a long-term benefit in the management of AD.[10]

## CONCLUSION

Both the public and the medical community have high hopes on exploring the RNAi pathway to treat incurable neurodegenerative diseases. The potency and selectivity of RNAi along with the ongoing advances in effective delivery of RNAi molecules lead us to be possibly optimistic about the potential of RNAi therapy reaching the neurology clinic. Early studies, both at the cellular and the animal models of neurodegenerative disease have provided encouraging results. But, the main concern is the safety of RNAi therapy in brain diseases, which is yet to be established. Further, efforts are to be made for an efficient and safe delivery of RNAi-mediating molecules into the target region of the brain. However, the rapid progress in the field of RNAi research indicates that in the next few years all these obstacles will vanish. The collaborative efforts of neuroscientists with RNAi researchers provided advancement in understanding the mechanism of action of endogenous miRNA pathway functions and its hope for gene therapists. We should salute the landmark discovery of Abdrew Fire etc. on RNAi, which gave a new hope that the treatment of neurodegenerative diseases through RNAi as a possible scenario. In the coming years, we will definitely observe the initial human clinical RNAi trials for neurodegenerative diseases such as amyotropic lateral sclerosis, Huntigton's disorder and the spino-cerebellar Ataxia. With joint efforts between academia and the biotechnology industry let us hope for the best in the service to neuropatients.
